# Diagnostic and Clinical Considerations in Myeloid Neoplasms with del(5q): Distinguishing Myelodysplastic Neoplasm with Low Blasts and del(5q) from Myeloid Neoplasms with del(5q) Post-Cytotoxic Therapy

**DOI:** 10.3390/genes17101255

**Published:** 2026-10-11

**Authors:** Changqing Xia, Zhenya Tang, Shanxiang Zhang, Allison Cushman-Vokoun, Krishna Gundabolu, Michael Haddadin, Joseph D. Khoury

**Affiliations:** 1 Department of Pathology, Microbiology and Immunology, University of Nebraska Medical Center, 42nd Street & Emile Street, Omaha, NE 68198, USA; ztang@unmc.edu (Z.T.); shazhang@unmc.edu (S.Z.); acushman@unmc.edu (A.C.-V.); jkhoury@unmc.edu (J.D.K.); 2 Division of Hematology, Department of Internal Medicine, University of Nebraska Medical Center, Omaha, NE 68198, USA; krishna.gundabolu@unmc.edu (K.G.); mihaddadin@unmc.edu (M.H.)

**Keywords:** del(5q), 5q− syndrome, MN-pCT, cytogenetics, *TP53*, haploinsufficiency, NAHR, NHEJ, genomic instability, clonal evolution, CHIP, cytogenetic test reporting

## Abstract

Deletion of the long arm of chromosome 5 is a defining cytogenetic abnormality in the myelodysplastic neoplasm with low blasts and isolated del(5q)(MDS-5q); however, it is also frequently observed in other myeloid neoplasms (MN), including MN post-cytotoxic therapy with del(5q) [(MN-pCT)-del(5q)]. Although isolated del(5q) has traditionally been considered indicative of de novo disease, emerging evidence demonstrates that similar cytogenetic findings may arise in biologically distinct contexts with markedly different clinical implications. In this review, we focus on the comparison between MDS-5q and MN-pCT-del(5q). Mechanistically, MDS-5q is primarily driven by the haploinsufficiency of genes within commonly deleted regions on chromosome 5q, including *RPS14* and regulatory *microRNAs*, resulting in lineage-restricted dysplasia and a relatively favorable prognosis, whereas del(5q) in MN-pCT-del(5q) typically arises from genotoxic stress and genomic instability and is often associated with complex karyotypes and adverse molecular features such as *TP53* mutations. Structural differences, including interstitial versus terminal deletions, may further reflect distinct underlying mechanisms of genomic rearrangement. A key advance is the recognition that del(5q) frequently occurs within a broader framework of clonal hematopoiesis of indeterminate potential (CHIP). CHIP-associated mutations in genes such as *DNMT3A*, *TET2*, and *ASXL1* are commonly identified and potentially precede or coexist with del(5q), thereby contributing to clonal fitness and disease evolution. Both “CHIP-first” and “del(5q)-first” evolutionary trajectories likely contribute to disease pathogenesis, underscoring the importance of clonal context in determining clinical behavior. In this framework, del(5q) is best characterized as a context-dependent event within dynamic clonal architecture, with important implications for risk stratification and therapeutic decision-making.

## 1. Introduction

Deletion of chromosome 5q (del(5q)) represents one of the most well-characterized cytogenetic abnormalities in hematologic malignancies and has historically served as a paradigm for linking chromosomal alterations to clinical phenotype [1,2]. The 5q− syndrome, first described by Van den Berghe and colleagues [2], is defined by a distinct constellation of clinical and morphologic features, including macrocytic anemia, hypolobated megakaryocytes, and a relatively favorable clinical course [2,3]. Over time, this entity has become a cornerstone in the classification of myelodysplastic neoplasms (MDS), with isolated del(5q) recognized as a defining cytogenetic abnormality in what is currently referred to as MDS with low blasts and del(5q) (MDS-5q) in the new classification guidelines from the World Health Organization and International Consensus Classification of myeloid neoplasms and acute leukemia [4,5,6]. The del (5q) is the one of the most common cytogenetic abnormalities in patients with MDSs. It has been reported that del(5q) occurs in 10–30% of patients with MDSs, and ~10% of cases are MDS-5q [7,8].

It has been demonstrated that del(5q) is more frequently observed in MN-arising post-cytotoxic therapy (MN-pCT-del(5q) (~42%)) compared to de novo MDSs and is often accompanied by monosomy 7 and/or del(7q) [9]. In this context, recent data suggest that del(5q) is a founding event that facilitates the evolution of a complex karyotype with a variety of numerical and structural alterations [10]. In such a heterogeneous group of disorders comprising MN-pCT-del(5q), chromosomal instability is caused by endogenous genomic damage or by exogenous exposure to cytotoxic chemotherapy, radiation therapy, or other genotoxic insults [9,11]. In these contexts, chromosome 5 abnormalities, often in conjunction with chromosome 7 alterations, are among the most common cytogenetic findings and are strongly associated with adverse outcomes [9,11,12].

The increasing use of cytotoxic therapies and improved cancer survival have led to a growing population of patients at risk for MN-pCT-del(5q), thereby increasing the frequency with which del(5q) may be encountered in a setting of clinical surveillance in patients with complex clinical problems. A particularly challenging scenario arises when del(5q) is identified as an isolated abnormality in a patient with prior therapy who might be carrying de novo del(5q). In such cases, it is of importance to distinguish classical MDS-5q from MN-pCT-del(5q), as this distinction has direct implications for prognosis and therapeutic decision-making, particularly regarding lenalidomide use [7].

However, solely relying on cytogenetic findings alone to make this distinction is problematic. Emerging evidence suggests that del(5q) is not a uniform biological entity, but rather reflects distinct mechanistic processes depending on clinical context, genomic architecture, and clonal evolution [10,13,14,15,16]. In this review, we examine the biological heterogeneity of del(5q), with particular emphasis on the biological and clinical differences between MDS-5q and MN-pCT-del(5q), as well as the cytogenetic diagnostic approach and reporting for such cases.

## 2. De Novo MDS-5q

**Figure 1:**
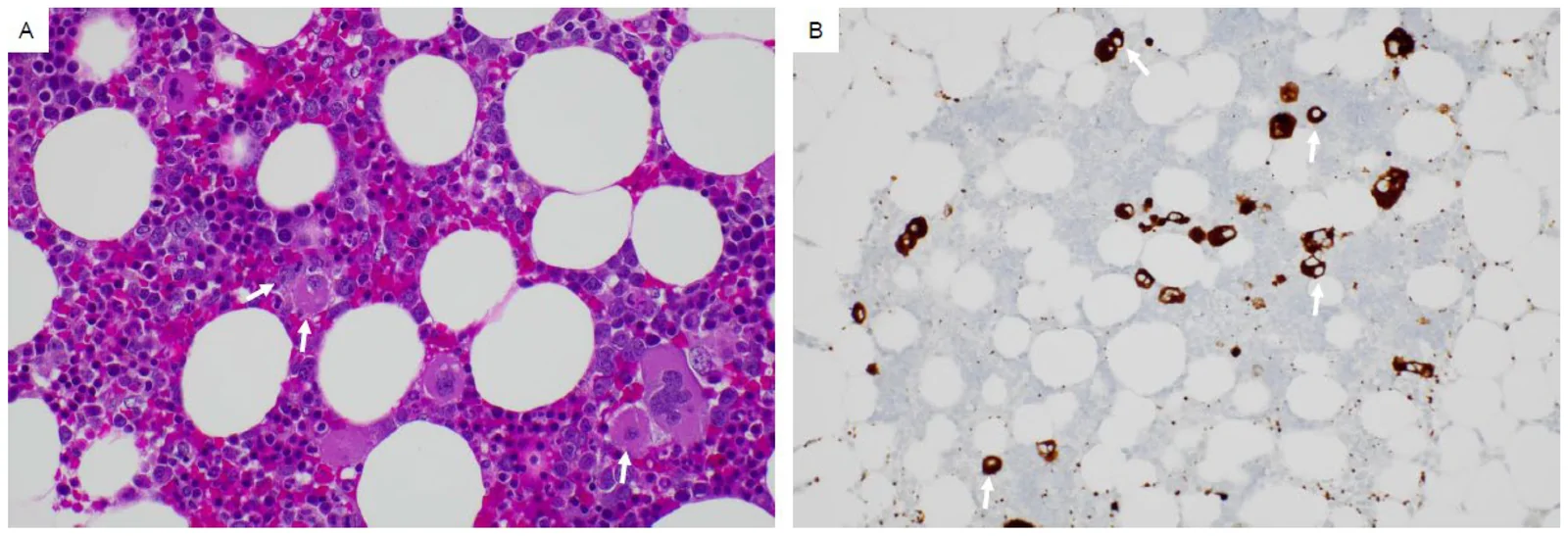
Hypolobated/monolobated megakaryocytes (white arrows) in MDS-5q. (**A**) Hematoxylin and eosin, 40× original amplification; (**B**) CD61 immunohistochemical stain, 20× original amplification.

As demonstrated in a large cohort study, MDS-5q is most often diagnosed in older adults (median age ~74 years), with female predominance (~3:1 F/M) [17], and typically presents with symptomatic anemia requiring transfusion support [1,2,18]. These demographic features are consistent with findings in the most recent large cohort study [18]. Platelet counts are often normal or elevated, and leukocyte counts are generally preserved. Bone marrow examination often reveals characteristic dysplastic changes predominantly affecting the megakaryocytic lineage, with hypolobated or monolobated megakaryocytes considered a hallmark feature [1,2] (Figure 1).

**Figure 2:**
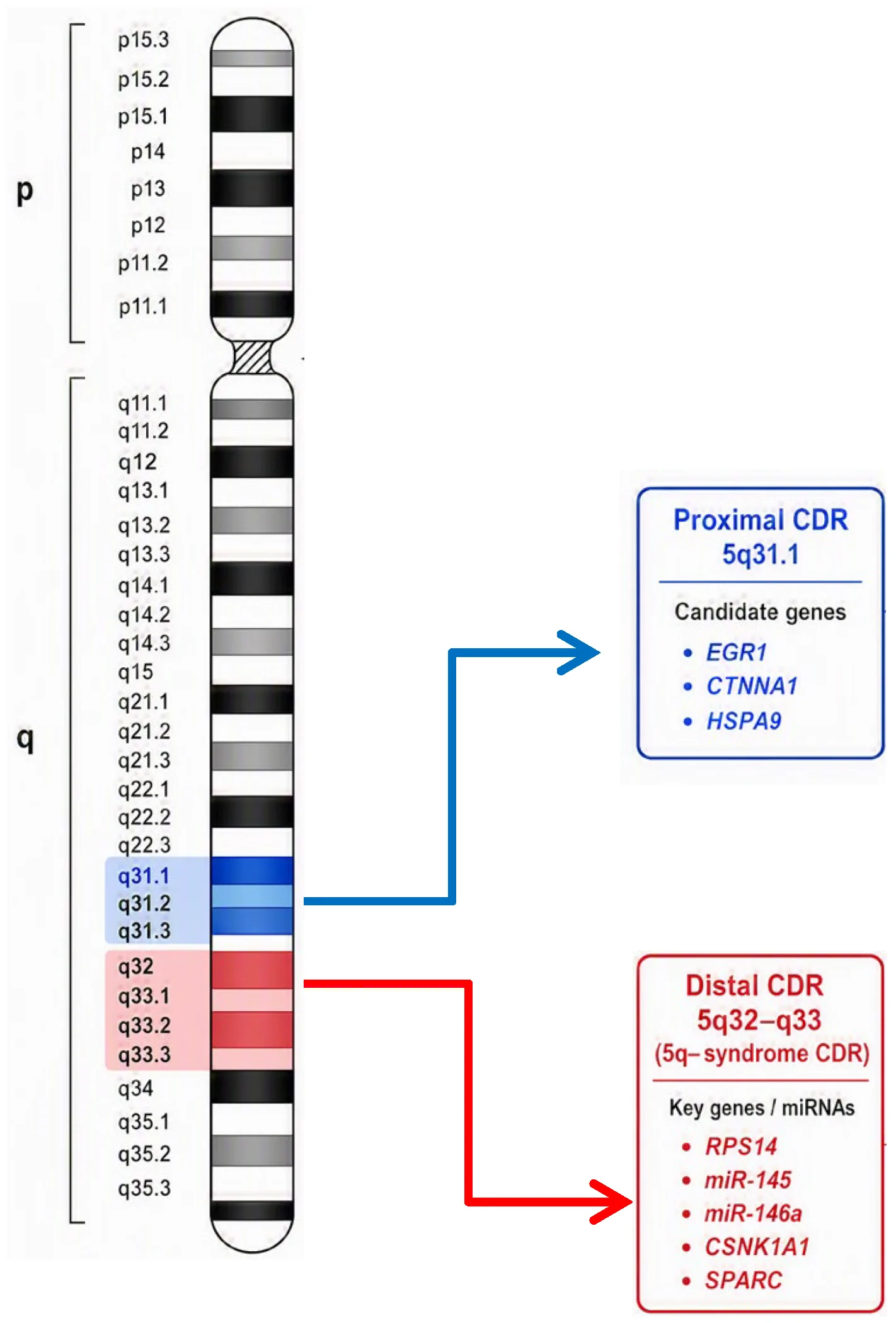
Common deleted regions (CDRs) of chromosome 5q in MDS-5q. An ISCN-style ideogram of chromosome 5 showing the proximal CDR at 5q31.1 (blue) and the distal CDR at 5q32q33 (red). The distal CDR is present in virtually all cases of MDS-5q, and contains *RPS14*, *MIR145*, *MIR146A*, *CSNK1A1* and *SPARK*. Haploinsufficiency of these genes contributes to the characteristic hematologic phenotype of this disease. The proximal CDR contains candidate tumor suppressor genes, including *EGR1*, *CTNNA1*, and *HSPA9*.

The clinical course of MDS-5q is generally indolent, with a relatively low risk of progression to AML compared to other MDS types [8,15,19]. Importantly, the introduction of lenalidomide has significantly improved outcomes in these patients, with high rates of transfusion independence and cytogenetic remission reported in clinical trials [7,8,20,21]. The mechanism of action of lenalidomide involves the cereblon-mediated degradation of casein kinase 1A1 (CK1α), a gene located within the commonly deleted region (CDR) of 5q (distal CDR) (Figure 2). Therefore, the haploinsufficient expression of CK1α renders neoplastic cells to be selectively sensitive to this effect [22,23,24]. However, patients with MDS-5q generally exhibit a poorer response to erythropoietin therapy than patients with other MDS types [25], likely due to the enhanced apoptosis of responding erythroid precursors resulted from haploinsufficiency of the genes within the deleted 5q region [13]. Despite its relatively favorable prognosis, heterogeneity exists across patients diagnosed with MDS-5q cases. MDS-5q, with one additional cytogenetic aberration except for del(7q) or monosomy 7, usually does not affect response to lenalidomide [13]. However, the presence of concurrent mutations, particularly in *TP53*, has been shown to adversely affect response to therapy and overall survival [26]. These findings underscore the importance of integrating molecular data into risk stratification.

## 3. MN-pCT-del(5q)

Therapy-related myeloid neoplasms is an umbrella term that encompasses MN-pCT-del(5q) characterized by prior exposure to cytotoxic therapy and a generally poor prognosis [9,11]. These neoplasms are typically associated with complex karyotypes and frequently involve simultaneous deletions or monosomies of chromosomes 5 and 7 [9,10,11,12].

The latency period between exposure and disease onset varies depending on the type of therapy, with alkylating agents and radiation typically associated with a latency of 5–10 years, whereas topoisomerase II inhibitors are associated with shorter latency periods [11]. The cytogenetic abnormalities observed in MN-pCT-del(5q) reflect the type of DNA damage induced by these agents. Importantly, although complex karyotypes are common, isolated del(5q) can be observed in early disease stages, even as part of clonal hematopoiesis (CH) or disease precursor preceding overt malignancy [27,28,29]. These cases may mimic MDS-5q both morphologically and cytogenetically (Figure 2), creating a diagnostic challenge. Moreover, MN-pCT-del(5q) cases are frequently associated with *TP53* mutations, which may be present even when cytogenetic findings appear simple [30,31].

## 4. Pathogenesis of MDS-5q and MN-pCT-del(5q)

### 4.1. Haploinsufficiency and Clonal Selection in MDS-5q

The pathogenic basis of MDS-5q lies in haploinsufficiency of multiple genes within the CDRs located on chromosome 5q31q33 [32,33,34] (Figure 2). Haploinsufficiency implies that loss of a single allele is sufficient to disrupt cellular function.

*RPS14* is a key gene within this region and plays a critical role in ribosomal biogenesis. *RPS14* haploinsufficiency leads to defective ribosome assembly, resulting in activation of the p53 pathway and impaired erythropoiesis [1,32]. This mechanism provides a direct link between the cytogenetic abnormality and the clinical phenotype of anemia.

In addition to *RPS14*, other genes within the CDRs contribute to disease pathogenesis. *miR-145* and *miR-146a* regulate innate immune signaling and megakaryocytic differentiation [33,35], while *EGR1* alongside *APC* located at 5q21 plays an important role in stem cell regulation and Wnt signaling [36,37]. The combined effect of these haploinsufficient genes results in a unique biological state in which hematopoietic stem cells gain a competitive advantage despite lineage-specific defects.

This process can be understood within the framework of clonal evolution, in which genetic alterations confer selective advantages that allow the expansion of specific clones [38,39]. In MDS-5q, the del(5q) clone likely represents an initiating event that establishes the dominant clone.

### 4.2. Segmental Duplications on Chromosome 5q Mediate Non-Allelic Homologous Recombination (NAHR) Underlying the Genetic Basis of MDS-5q

The genomic architecture of chromosome 5q may predispose to deletion formation through the presence of segmental duplications [40,41,42,43,44]. These regions of high sequence homology can facilitate NAHR, a mechanism in which recombination occurs between non-allelic homologous sequences, resulting in deletion of the intervening region.

NAHR has been well characterized in constitutional genomic disorders and is increasingly recognized in somatic rearrangements as well [42,45,46]. In the context of del(5q), the presence of recurrent deletion boundaries suggests that genomic architecture may influence breakpoint localization. However, unlike classical NAHR-mediated syndromes, del(5q) exhibits variability in deletion size and breakpoints [47,48] (Figure 2), indicating that multiple mechanisms may be involved.

Alternative mechanisms, such as microhomology-mediated end joining (MMEJ) and replication-based processes, may also contribute to deletion formation [45]. Thus, NAHR may represent one component of a broader spectrum of mechanisms contributing to interstitial deletions in MDS-5q.

### 4.3. DNA Damage and Non-Homologous End Joining (NHEJ) in MN-pCT-del(5q)

In MN-pCT-del(5q), del(5q) is more likely to arise from DNA damage induced by cytotoxic agents. Double-strand breaks represent a critical form of DNA damage and are repaired through pathways such as homologous recombination and NHEJ [49,50].

NHEJ is an error-prone repair mechanism that directly ligates DNA ends without requiring extensive sequence homology. This process can result in deletions and chromosomal rearrangements, particularly in the setting of extensive DNA damage [51,52,53]. In MN-pCT-del(5q), repeated exposure to cytotoxic agents induces DNA damage and promotes chromosomal instability through defective DNA repair mechanisms [54]. As a result, large deletions involving chromosome 5q frequently arise as part of complex karyotypes, reflecting the progressive accumulation of cytogenetic abnormalities [14]. *TP53* mutations are highly enriched in MN-pCT, especially in cases with complex cytogenetic alterations, including those with del(5q) [14,55]. Loss of *TP53* function disrupts DNA damage response pathways and apoptosis, enabling the survival and expansion of cells harboring extensive genomic damage and further promoting clonal evolution [56].

## 5. Structural Spectrum of del(5q) Aberrations in MDS-5q and MN-pCT-del(5q)

### 5.1. Interstitial Deletions

Interstitial deletions of 5q involve two breakpoints within the long arm of chromosome 5, resulting in the removal of an internal segment while preserving telomeric sequences which is critical for maintaining chromosome stability. These deletions are characteristic of MDS-5q, which is defined by an isolated interstitial del(5q). Moreover, these deletions typically localize to well-defined CDRs, particularly within bands 5q31q33 (Figure 2), which harbor genes critical to disease pathogenesis [1,57]. The relative consistency of interstitial deletions suggests that they may arise through the aforementioned recombination-based mechanisms and may be subject to selective pressures favoring specific genomic configurations.

### 5.2. Terminal Deletions

Terminal deletions involve a single breakpoint and result in the loss of the distal chromosome arm. These deletions are more commonly observed in MN-pCT-del(5q) [9,58] and are often associated with telomere dysfunction [59,60,61].

Loss of telomeres can lead to breakage–fusion–bridge cycles, a process in which chromosomes fuse and break during cell division, generating additional genomic instability [59]. This mechanism contributes to clonal heterogeneity and disease progression.

Collectively, the structural configuration of del(5q) aberrations may reflect underlying mechanisms and influence disease behavior. Whereas interstitial deletions are generally more stable and may reflect selective processes, terminal deletions are associated with genomic instability and may indicate a damage-driven pathogenic process [45,62].

## 6. Molecular Context and Clonal Evolution

### 6.1. TP53 Mutations and Genomic Instability

The molecular landscape in which del(5q) arises plays a critical role in shaping disease phenotype, progression, and therapeutic response. Among the various genetic alterations associated with myeloid neoplasms, *TP53* mutations are of particular importance. The protein p53, a product of the *TP53* gene, functions as a central regulator of genomic integrity, coordinating cellular responses to DNA damage through the induction of cell cycle arrest, senescence, or apoptosis [31,50]. Loss of *TP53* function allows cells harboring substantial genomic abnormalities, including large chromosomal deletions, to evade apoptosis and continue proliferating.

In the context of del(5q), *TP53* mutations are highly enriched in MN-pCT-del(5q) cases, especially in those with complex karyotypes [31]. Notably, *TP53* mutations may be present even in cases with apparently isolated del(5q) [63], suggesting that cytogenetic simplicity does not necessarily equate to biological indolence. Interestingly, *TP53* has been documented as a CHIP-associated driver gene [27,28,48]. This possibility should be considered particularly when *TP53* mutations are detected at low-variant-allele frequency (VAF) in the absence of other features supporting a dominant disease-associated clone [27,28,48]. Studies have also demonstrated that *TP53*-mutated clones often pre-exist at low VAF in MN and undergo expansion under selective pressure from cytotoxic therapy, ultimately giving rise to overt disease [31,64]. This model provides a mechanistic link between CHIP, prior therapy, genomic instability, clonal evolution and chemotherapy-resistant clonal expansion.

Clinically, the presence of *TP53* mutations is associated with resistance to conventional therapies, including lenalidomide, and with poor overall survival [65,66]. In contrast to lenalidomide, pomalidomide treatment did not provide an equivalent level of selective advantage to *TP53*-mutant HSPCs, providing a biological rationale for its use in patients at high risk for MN-pCT-del(5q) [66]. Thus, assessment of *TP53* status has become an essential component of the diagnostic workup in patients with del(5q), particularly in those with a history of prior therapy.

### 6.2. Co-Mutations and Clonal Architecture

Beyond *TP53*, a wide spectrum of co-occurring mutations contributes to the molecular heterogeneity of del(5q)-associated neoplasms. Frequently mutated genes include those involved in epigenetic regulation (e.g., *DNMT3A*, *TET2*, *ASXL1*), RNA splicing (e.g., *SF3B1*, *SRSF2*), and transcriptional control (e.g., *RUNX1*) [26,67,68,69,70,71]. These mutations often arise in HSPCs and may precede the acquisition of del(5q), particularly in the setting of age-related CH [27,28,72].

Clonal evolution studies using deep sequencing approaches have demonstrated that del(5q) may represent either an early/founding lesion that establishes the dominant clone or a secondary cytogenetic event acquired during disease progression, depending on the underlying molecular context and co-occurring mutations [14,15,73]. In classical MDS-5q cases, del(5q) is typically an early driver lesion that defines the dominant clone and shapes the clinical phenotype. In contrast, in MN-pCT-del(5q) cases, del(5q) may be acquired on a background of pre-existing CH, representing a later event that contributes to disease progression.

The interplay between del(5q) and co-mutations influences not only disease biology but also therapeutic response. For example, mutations in genes involved in epigenetic regulation may modulate sensitivity to hypomethylating agents, whereas *TP53* mutations confer resistance to multiple therapeutic modalities. Understanding the clonal architecture and temporal sequence of genetic events is therefore essential for accurate risk stratification and for the development of personalized treatment strategies.

### 6.3. CH and CHIP-Associated Mutations in del(5q)

The recognition of CHIP has reshaped our understanding of myeloid neoplasia, including the pathogenesis of MN with del(5q). CHIP is defined by the presence of somatic mutations in hematopoietic cells among individuals without evidence of overt hematologic malignancy. CHIP prevalence increases significantly with age [27,28,72,74]. Given that MDS-5q predominantly affects older adults, it is not unexpected that CHIP-associated mutations frequently coexist with del(5q). However, as described above, “del(5q) first” or “CHIP first” may demonstrate distinct disease processes and progression.

Recent sequencing studies have demonstrated that approximately 30–60% of patients with isolated del(5q) harbor at least one additional somatic mutation, many of which fall within the spectrum of CHIP-associated genes [18,26,67,68,69,70,71]. Among these, *DNMT3A*, *TET2*, and *ASXL1* are the most affected, with variant allele frequencies (VAF) typically ranging from 10 to 20% for *DNMT3A* and *TET2*, and somewhat lower for *ASXL1*. Mutations in *SF3B1* and other splicing factors are also often observed, further contributing to molecular heterogeneity [67,70].

An important question concerns the temporal relationship between CHIP mutations and del(5q). Accumulating evidence supports a model in which CHIP-associated mutations often represent early or pre-leukemic events, occurring prior to acquisition of cytogenetic abnormalities [27,28,72]. In this framework, a hematopoietic stem cell harboring a CHIP mutation expands to form a clonal population, which subsequently acquires del(5q) as a secondary event during clonal evolution. This model is supported by VAF-based clonal hierarchy analyses demonstrating that CHIP-associated mutations can be present at higher allele burdens than the del(5q) cytogenetic clone, as well as by longitudinal studies showing the persistence of CHIP clones even when del(5q) clones are suppressed by therapy [13,63,75,76].

However, the reverse sequence where del(5q) arises as the founding lesion followed by the acquisition of additional mutations likely also occurs, particularly in classical MDS-5q with otherwise “clean” genomic profiles [14,15,77]. Thus, the relationship between CHIP mutations and del(5q) is best viewed as context-dependent and bidirectional, reflecting the dynamic nature of clonal evolution.

Functionally, CHIP-associated mutations may cooperate with the haploinsufficiency of genes within the 5q CDRs to promote clonal fitness. For example, mutations in *DNMT3A* and *TET2* alter epigenetic regulation and enhance the self-renewal of hematopoietic stem cells, potentially providing a permissive environment for the expansion of del(5q) clones [26,67,68,69]. Similarly, the disruption of immune signaling pathways by *TET2* or *ASXL1* mutations may synergize with *miR-146a* haploinsufficiency, further contributing to dysregulated hematopoiesis [35,78,79,80,81].

Importantly, the presence of CHIP-associated mutations may influence clinical phenotype and therapeutic response. While classical MDS-5q is typically associated with favorable prognosis and robust response to lenalidomide, co-occurring mutations, particularly in high-risk genes such as *TP53*, can attenuate these responses and confer a higher risk of disease progression [18,65]. Thus, incorporation of mutational profiling into the evaluation of del(5q) is essential for accurate risk stratification.

Overall, these observations support a model in which MDS-5q represents not a purely single-lesion disorder but rather a composite clonal architecture, in which haploinsufficiency-driven biology is modulated by a variable background of CHIP-associated mutations. A comparison of CHIP-associated mutations in MDS-5q vs. MN-pCT-del(5q) is listed in Table 1 [26,67,68,69,70,71].

## 7. Limitations of Examining del(5q) by Conventional Cytogenetic Testing

Despite its central role in the diagnosis and classification of myeloid neoplasms, conventional cytogenetic analysis has important limitations that constrain its ability to fully characterize del(5q) and its biological significance. These limitations become particularly evident when cytogenetic findings are interpreted in isolation, without the integration of clinical and molecular data.

First, conventional G-banded karyotyping has an effective resolution of approximately 5–10 Mb, depending on chromosomal banding quality and the stage of chromosome condensation (ISCN—Interpretation and communication of human cytogenomic nomenclature/Home). As a result, smaller deletions and duplications, cryptic rearrangements or complex structural alterations involving 5q may not be identified. Fluorescence in situ hybridization (FISH) improves the sensitivity for detecting del(5q), particularly in cases with low-level clones or poor metaphase yield; however, FISH is locus-specific and does not provide comprehensive information regarding deletion size, breakpoint location, or overall genomic architecture. Consequently, both karyotype and FISH may fail to distinguish between interstitial and terminal deletions in borderline cases or may underestimate underlying structural complexity.

Second, cytogenetic analysis does not provide direct insight into the mechanism of deletion formation. As discussed above, interstitial deletions may arise through recombination-based mechanisms such as NAHR, whereas terminal deletions are more consistent with DNA damage and error-prone repair processes such as NHEJ [42,45,46,49,53]. However, these mechanistic distinctions cannot be definitively inferred from cytogenetic data alone. Without breakpoint-level resolution, it is difficult to determine whether a deletion reflects a targeted, recurrent event or a stochastic consequence of genomic instability.

Third, cytogenetics lacks the ability to assess the molecular context in which del(5q) occurs. The presence or absence of co-occurring mutations, particularly in genes such as *TP53*, *DNMT3A*, *TET2* or *ASXL1*, has profound implications for disease classification, prognosis, and therapeutic response [26,67,68,69]. For example, *TP53* mutations are strongly associated with therapy-related disease and adverse outcomes, even in cases with apparently simple cytogenetic findings [31]. Thus, reliance on cytogenetics alone may lead to an underestimation of disease risk.

Fourth, cytogenetic findings do not capture the temporal dynamics of clonal evolution. The change of del(5q) may represent a founding event in MDS-5q or a secondary event in MN-pCT-del(5q) arising within a pre-existing CH clone [28,72]. Without longitudinal sampling or molecular phylogenetic analysis, it is often impossible to determine the chronological order of genetic events. This distinction is clinically relevant, as early founder events may define disease phenotype, whereas secondary events may reflect disease progression.

Finally, cytogenetic interpretation is inherently dependent on clinical context, which may not always be fully available to the laboratory. Information regarding prior chemotherapy or radiation exposure, latency period, and clinical presentation is essential for distinguishing de novo from therapy-related disease [9,11,82]. In the absence of such information, cytogenetic findings may be misinterpreted and/or misapplied, leading to inappropriate classification or management.

Taken together, these limitations underscore the need for an integrative diagnostic approach that combines cytogenetic, molecular, and clinical data. Advances in genomic technologies, including chromosomal microarray, optical genome mapping and next-generation sequencing, provide higher-resolution insights into genomic structure and mutational landscape and are increasingly essential for the accurate interpretation of del(5q) in modern clinical practice [67,68,70,71]. It is worth noting that emerging long-read sequencing technologies have shown considerable diagnostic value in hematologic malignancies, including MDS [83,84]. These approaches not only facilitate the high-resolution characterization of structural genomic alterations but also enable the detection of clinically relevant gene mutations that may play important roles in disease pathogenesis.

## 8. Integrative Diagnostic Approach

Given the limitations of cytogenetic analysis alone, an integrative approach is essential for the accurate interpretation of del(5q) as an apparently sole chromosomal aberration. Such an approach should systematically incorporate cytogenetic findings, clinical history, morphologic assessment, and molecular profiling to arrive at a biologically and clinically meaningful classification.

We propose a stepwise framework as described below:

**Step 1: Confirmation of del(5q) and assessment of cytogenetic complexity**

The initial step involves confirming the presence of del(5q) using conventional karyotyping and/or FISH. Whenever possible, karyotype analysis should be performed to assess the global chromosomal landscape, including the presence of additional abnormalities. FISH can be used to confirm the deletion and to detect subclonal populations. Even when del(5q) appears isolated, careful evaluation for subtle and subclonal abnormalities is warranted. The presence of additional cytogenetic changes, even at low levels, may indicate underlying genomic instability and support a therapy-related origin [19,85,86].

**Step 2: Clinical context assessment**

A detailed clinical history is critical. Attention should be paid to prior exposure to cytotoxic chemotherapy, radiation therapy, or other genotoxic agents. The type of therapy and latency period may provide clues regarding the likelihood of therapy-related disease [11]. For example, a latency of several years following alkylating agent exposure strongly suggests a therapy-related etiology.

**Step 3: Morphologic evaluation**

Bone marrow morphology should be carefully reviewed. The presence of characteristic hypolobated megakaryocytes supports a diagnosis of classical MDS-5q, whereas multilineage dysplasia, increased blasts, or marked cytopenias may favor a more-aggressive or therapy-related process. However, morphologic features are not entirely specific and must be interpreted in conjunction with other data.

**Step 4: Molecular profiling**

NGS plays a pivotal role in modern diagnostics. Particular emphasis should be placed on assessing *TP53* mutation status, given its strong association with therapy-related disease and poor prognosis [65]. The presence of additional mutations in genes such as *ASXL1*, *RUNX1*, or *DNMT3A* may further refine risk stratification [26,67,68,69].

**Step 5: Integration and classification**

Finally, all available data should be integrated to arrive at a final classification. In cases with no prior therapy, characteristic morphology, absence of high-risk mutations, and isolated interstitial del(5q), a diagnosis of classical MDS-5q is appropriate. In contrast, cases with prior cytotoxic exposure, *TP53* mutation, or evidence of genomic instability should be considered therapy-related, even if cytogenetic findings appear simple.

Importantly, this framework emphasizes that no single parameter is definitive. Rather, the diagnosis emerges from the convergence of multiple lines of evidence. This integrative approach aligns with current WHO and ICC recommendations, which increasingly emphasize the incorporation of molecular and clinical data into disease classification [4,5]. Nevertheless, implementing this integrative approach may be challenging for reference laboratories with limited access to pathologists and clinicians. The following reporting strategy may help mitigate certain aspects of this challenge.

## 9. Implications for Cytogenetic Reporting

The evolving understanding of del(5q) has important implications for how cytogenetic findings are reported in clinical practice. Traditionally, cytogenetic reports have focused on descriptive findings, with limited interpretation and clinical implication. However, given the clinical significance of del(5q), there is increasing demand for interpretive comments that assist pathologists and clinicians in diagnosis and management.

A key principle in reporting is to avoid overinterpretation. Cytogenetic findings alone are insufficient to distinguish between MDS-5q and MN-pCT-del(5q), and reports should clearly communicate this limitation. Overly definitive statements based solely on cytogenetic findings may lead to misclassification and inappropriate treatment decisions.

Instead, reports should adopt standardized language that emphasizes the need for clinical and molecular correlation. For example: “An isolated deletion of chromosome 5q is identified. This finding is characteristic of MDS-5q in the appropriate clinical context; however, similar abnormalities may also occur in MN-pCT-del(5q) or other types of MN. Correlation with clinical history, morphologic findings, and molecular studies (including *TP53* mutation status) is recommended.”

In addition, reports may include comments on the structural nature of the deletion, when discernible, as this may provide indirect insight into underlying mechanisms. For instance, identification of a large terminal deletion may raise suspicion for a damage-related process, whereas a well-defined interstitial deletion may be more consistent with classical disease. However, such interpretations should be presented cautiously and framed as hypotheses rather than definitive conclusions.

Another important consideration is the communication of prognostic implications. While isolated del(5q) is generally associated with favorable prognosis, this may not apply in the presence of high-risk molecular features or therapy-related context [9,26,48,63,69]. Cytogenetic reports should therefore avoid blanket prognostic statements and instead highlight factors that may modify risk.

Finally, effective reporting benefits from close collaboration among cytogeneticists, molecular geneticists, hematopathologists, and hematologic oncologists. Although such collaboration may not always be readily available in all practice settings, multidisciplinary discussions, particularly through tumor boards or case conferences, should be considered whenever it is feasible to arrive at more accurate and nuanced interpretations.

## 10. Conclusions

Deletion of chromosome 5q is a clinically distinctive cytogenetic abnormality in MN, but it does not define a single disease entity. Instead, del(5q) represents a spectrum of biologically and clinically diverse states shaped by molecular context and origin. Classical MDS-5q reflects the haploinsufficiency of genes within CDRs, including *RPS14* and regulatory *microRNAs*, producing a relatively stable phenotype with lineage-restricted dysplasia and favorable treatment response. In contrast, MN-pCT-del(5q) arises under genotoxic stress, frequently co-occurs with adverse alterations such as *TP53* mutations, and is associated with poor clinical outcomes.

An important insight is that del(5q) often develops within pre-existing CH. Commonly observed mutations in genes such as *DNMT3A*, *TET2*, and *ASXL1* indicate that many cases emerge from or alongside established mutant clones rather than from normal hematopoietic stem cells. These evolutionary pathways are diverse and overlapping, emphasizing that the clinical significance of del(5q) depends on its clonal context. This has key diagnostic and therapeutic implications. Cytogenetics alone cannot distinguish MDS-5q from MN-pCT-del(5q). Accurate classification requires integration of clinical history, morphology, and comprehensive molecular profiling, including *TP53* status. Such an approach is critical for risk stratification and predicting response to therapies such as lenalidomide.

Future advances in genomic profiling and clonal tracking will refine del(5q) classification, shifting from static definitions toward models incorporating clonal architecture and evolution, ultimately improving personalized patient management.

## Figures and Tables

**Table 1:** Comparison of CHIP-associated mutations in MDS-5q vs. MN-pCT-del(5q) [26,67,68,69,70,71].

Features	MDS-5q	MN-pCT-del(5q)
Overall mutation frequency	~30–60%	~60–90% (often higher)
CHIP gene mutations (*DNMT3A*, *TET2*, *ASXL1*)	Common (~20–40% combined)	Very common (~30–50% combined)
*DNMT3A*	~10–20%	~15–25%
*TET2*	~10–20%	~15–25%
*ASXL1*	~5–15%	~10–20%
*TP53*	~5–20% (enriched in progressed cases)	Very frequent (~30–50%)
Clonal timing	Often early (CHIP-first) or co-dominant	Frequently pre-existing CHIP clone expanded by therapy
Clonal architecture	Relatively simple; fewer subclones	Complex; multiple subclones common
Functional role	Modulates haploinsufficiency-driven phenotype	Drives genomic instability and disease progression
Impact on therapy	May slightly modify lenalidomide response	Strongly associated with poor response and resistance

## Data Availability

No new data were created or analyzed in this study. Data sharing is not applicable to this article.

## References

[1] Boultwood J., Pellagatti A., McKenzie A.N., Wainscoat J.S. (2010). Advances in the 5q-syndrome. Blood.

[2] Van den Berghe H., Cassiman J.J., David G., Fryns J.P., Michaux J.L., Sokal G. (1974). Distinct haematological disorder with deletion of long arm of no. 5 chromosome. Nature.

[3] Tefferi A., Mathew P., Noel P. (1994). The 5q-syndrome: A scientific and clinical update. Leuk. Lymphoma.

[4] Khoury J.D., Solary E., Abla O., Akkari Y., Alaggio R., Apperley J.F., Bejar R., Berti E., Busque L., Chan J.K.C. (2022). The 5th edition of the World Health Organization Classification of Haematolymphoid Tumours: Myeloid and Histiocytic/Dendritic Neoplasms. Leukemia.

[5] Arber D.A., Orazi A., Hasserjian R.P., Borowitz M.J., Calvo K.R., Kvasnicka H.M., Wang S.A., Bagg A., Barbui T., Branford S. (2022). International Consensus Classification of Myeloid Neoplasms and Acute Leukemias: Integrating morphologic, clinical, and genomic data. Blood.

[6] Cazzola M., Sehn L.H. (2022). Developing a classification of hematologic neoplasms in the era of precision medicine. Blood.

[7] List A., Dewald G., Bennett J., Giagounidis A., Raza A., Feldman E., Powell B., Greenberg P., Thomas D., Stone R. (2006). Lenalidomide in the myelodysplastic syndrome with chromosome 5q deletion. N. Engl. J. Med..

[8] List A.F., Bennett J.M., Sekeres M.A., Skikne B., Fu T., Shammo J.M., Nimer S.D., Knight R.D., Giagounidis A., Investigators M.D.S.S. (2014). Extended survival and reduced risk of AML progression in erythroid-responsive lenalidomide-treated patients with lower-risk del(5q) MDS. Leukemia.

[9] Smith S.M., Le Beau M.M., Huo D., Karrison T., Sobecks R.M., Anastasi J., Vardiman J.W., Rowley J.D., Larson R.A. (2003). Clinical-cytogenetic associations in 306 patients with therapy-related myelodysplasia and myeloid leukemia: The University of Chicago series. Blood.

[10] Creamer J.P., Ray S., Stewart S., Gulsuner S., Saliba A.N., Wu J., Huang F.Y., Leppa A.M., Wang B., Abbas H.A. (2026). Chromosome 5q deletion drives evolution of aneuploidy in myeloid neoplasms with complex karyotype. Blood.

[11] Godley L.A., Larson R.A. (2008). Therapy-related myeloid leukemia. Semin. Oncol..

[12] Leone G., Pagano L., Ben-Yehuda D., Voso M.T. (2007). Therapy-related leukemia and myelodysplasia: Susceptibility and incidence. Haematologica.

[13] Roncador M., Bernard E., Hasserjian R., Boultwood J., Elena C., Galli A., Gurnari C., Mecucci C., Michaux L., Mittelman M. (2025). A precision medicine approach to the myelodysplastic syndrome with isolated deletion 5q, 50 years after its discovery. Blood.

[14] Huber S., Hutter S., Baer C., Meggendorfer M., Hoermann G., Kern W., Haferlach T., Haferlach C. (2025). Two ways to complex karyotype in MDS-the role of del(5q) and TP53. Blood Cancer J..

[15] Adema V., Palomo L., Walter W., Mallo M., Hutter S., La Framboise T., Arenillas L., Meggendorfer M., Radivoyevitch T., Xicoy B. (2022). Pathophysiologic and clinical implications of molecular profiles resultant from deletion 5q. EBioMedicine.

[16] Acha P., Mallo M., Sole F. (2022). Myelodysplastic Syndromes with Isolated del(5q): Value of Molecular Alterations for Diagnostic and Prognostic Assessment. Cancers.

[17] Patnaik M.M., Lasho T.L., Finke C.M., Gangat N., Caramazza D., Holtan S.G., Pardanani A., Knudson R.A., Ketterling R.P., Chen D. (2010). WHO-defined ‘myelodysplastic syndrome with isolated del(5q)’ in 88 consecutive patients: Survival data, leukemic transformation rates and prevalence of JAK2, MPL and IDH mutations. Leukemia.

[18] Giagounidis A.A., Germing U., Wainscoat J.S., Boultwood J., Aul C. (2004). The 5q-syndrome. Hematology.

[19] Germing U., Lauseker M., Hildebrandt B., Symeonidis A., Cermak J., Fenaux P., Kelaidi C., Pfeilstocker M., Nosslinger T., Sekeres M. (2012). Survival, prognostic factors and rates of leukemic transformation in 381 untreated patients with MDS and del(5q): A multicenter study. Leukemia.

[20] Fenaux P., Giagounidis A., Selleslag D., Beyne-Rauzy O., Mufti G., Mittelman M., Muus P., Te Boekhorst P., Sanz G., Del Canizo C. (2011). A randomized phase 3 study of lenalidomide versus placebo in RBC transfusion-dependent patients with Low-/Intermediate-1-risk myelodysplastic syndromes with del5q. Blood.

[21] Giagounidis A., Mufti G.J., Mittelman M., Sanz G., Platzbecker U., Muus P., Selleslag D., Beyne-Rauzy O., Te Boekhorst P., del Canizo C. (2014). Outcomes in RBC transfusion-dependent patients with Low-/Intermediate-1-risk myelodysplastic syndromes with isolated deletion 5q treated with lenalidomide: A subset analysis from the MDS-004 study. Eur. J. Haematol..

[22] Kronke J., Fink E.C., Hollenbach P.W., MacBeth K.J., Hurst S.N., Udeshi N.D., Chamberlain P.P., Mani D.R., Man H.W., Gandhi A.K. (2015). Lenalidomide induces ubiquitination and degradation of CK1alpha in del(5q) MDS. Nature.

[23] Fink E.C., Ebert B.L. (2015). The novel mechanism of lenalidomide activity. Blood.

[24] Petzold G., Fischer E.S., Thoma N.H. (2016). Structural basis of lenalidomide-induced CK1alpha degradation by the CRL4(CRBN) ubiquitin ligase. Nature.

[25] Kelaidi C., Park S., Brechignac S., Mannone L., Vey N., Dombret H., Aljassem L., Stamatoullas A., Ades L., Giraudier S. (2008). Treatment of myelodysplastic syndromes with 5q deletion before the lenalidomide era; the GFM experience with EPO and thalidomide. Leuk. Res..

[26] Bejar R., Stevenson K., Abdel-Wahab O., Galili N., Nilsson B., Garcia-Manero G., Kantarjian H., Raza A., Levine R.L., Neuberg D. (2011). Clinical effect of point mutations in myelodysplastic syndromes. N. Engl. J. Med..

[27] Genovese G., Kahler A.K., Handsaker R.E., Lindberg J., Rose S.A., Bakhoum S.F., Chambert K., Mick E., Neale B.M., Fromer M. (2014). Clonal hematopoiesis and blood-cancer risk inferred from blood DNA sequence. N. Engl. J. Med..

[28] Jaiswal S., Fontanillas P., Flannick J., Manning A., Grauman P.V., Mar B.G., Lindsley R.C., Mermel C.H., Burtt N., Chavez A. (2014). Age-related clonal hematopoiesis associated with adverse outcomes. N. Engl. J. Med..

[29] Voso M.T., Falconi G., Fabiani E. (2021). What’s new in the pathogenesis and treatment of therapy-related myeloid neoplasms. Blood.

[30] Bao Z., Li B., Qin T., Xu Z., Qu S., Jia Y., Li C., Pan L., Gao Q., Jiao M. (2025). Molecular characteristics and clinical implications of TP53 mutations in therapy-related myelodysplastic syndromes. Blood Cancer J..

[31] Wong T.N., Ramsingh G., Young A.L., Miller C.A., Touma W., Welch J.S., Lamprecht T.L., Shen D., Hundal J., Fulton R.S. (2015). Role of TP53 mutations in the origin and evolution of therapy-related acute myeloid leukaemia. Nature.

[32] Ebert B.L., Pretz J., Bosco J., Chang C.Y., Tamayo P., Galili N., Raza A., Root D.E., Attar E., Ellis S.R. (2008). Identification of RPS14 as a 5q-syndrome gene by RNA interference screen. Nature.

[33] Starczynowski D.T., Kuchenbauer F., Argiropoulos B., Sung S., Morin R., Muranyi A., Hirst M., Hogge D., Marra M., Wells R.A. (2010). Identification of miR-145 and miR-146a as mediators of the 5q-syndrome phenotype. Nat. Med..

[34] Boultwood J., Pellagatti A., Cattan H., Lawrie C.H., Giagounidis A., Malcovati L., Della Porta M.G., Jadersten M., Killick S., Fidler C. (2007). Gene expression profiling of CD34+ cells in patients with the 5q-syndrome. Br. J. Haematol..

[35] Ribezzo F., Snoeren I.A.M., Ziegler S., Stoelben J., Olofsen P.A., Henic A., Ferreira M.V., Chen S., Stalmann U.S.A., Buesche G. (2019). Rps14, Csnk1a1 and miRNA145/miRNA146a deficiency cooperate in the clinical phenotype and activation of the innate immune system in the 5q-syndrome. Leukemia.

[36] Stoddart A., Fernald A.A., Wang J., Davis E.M., Karrison T., Anastasi J., Le Beau M.M. (2014). Haploinsufficiency of del(5q) genes, Egr1 and Apc, cooperate with Tp53 loss to induce acute myeloid leukemia in mice. Blood.

[37] Stoddart A., Wang J., Fernald A.A., Karrison T., Anastasi J., Le Beau M.M. (2014). Cell intrinsic and extrinsic factors synergize in mice with haploinsufficiency for Tp53, and two human del(5q) genes, Egr1 and Apc. Blood.

[38] Greaves M., Maley C.C. (2012). Clonal evolution in cancer. Nature.

[39] McGranahan N., Swanton C. (2017). Clonal Heterogeneity and Tumor Evolution: Past, Present, and the Future. Cell.

[40] Bailey J.A., Eichler E.E. (2006). Primate segmental duplications: Crucibles of evolution, diversity and disease. Nat. Rev. Genet..

[41] Sharp A.J., Hansen S., Selzer R.R., Cheng Z., Regan R., Hurst J.A., Stewart H., Price S.M., Blair E., Hennekam R.C. (2006). Discovery of previously unidentified genomic disorders from the duplication architecture of the human genome. Nat. Genet.

[42] Zhang F., Gu W., Hurles M.E., Lupski J.R. (2009). Copy number variation in human health, disease, and evolution. Annu. Rev. Genom. Hum. Genet.

[43] Buysse K., Crepel A., Menten B., Pattyn F., Antonacci F., Veltman J.A., Larsen L.A., Tumer Z., de Klein A., van de Laar I. (2008). Mapping of 5q35 chromosomal rearrangements within a genomically unstable region. J. Med. Genet.

[44] Courseaux A., Richard F., Grosgeorge J., Ortola C., Viale A., Turc-Carel C., Dutrillaux B., Gaudray P., Nahon J.L. (2003). Segmental duplications in euchromatic regions of human chromosome 5: A source of evolutionary instability and transcriptional innovation. Genome Res..

[45] Hastings P.J., Lupski J.R., Rosenberg S.M., Ira G. (2009). Mechanisms of change in gene copy number. Nat. Rev. Genet.

[46] Carvalho C.M., Lupski J.R. (2016). Mechanisms underlying structural variant formation in genomic disorders. Nat. Rev. Genet.

[47] Nofrini V., La Starza R., Crescenzi B., Pierini V., Barba G., Mecucci C. (2012). Different boundaries characterize isolated and non-isolated 5q deletions in myelodysplastic syndromes and acute myeloid leukemias. Haematologica.

[48] Santana-Davila R., Holtan S.G., Dewald G.W., Ketterling R.P., Knudson R.A., Hanson C.A., Steensma D.P., Tefferi A. (2008). Chromosome 5q deletion: Specific diagnoses and cytogenetic details among 358 consecutive cases from a single institution. Leuk. Res..

[49] Lieber M.R. (2010). The mechanism of double-strand DNA break repair by the nonhomologous DNA end-joining pathway. Annu. Rev. Biochem..

[50] Jackson S.P., Bartek J. (2009). The DNA-damage response in human biology and disease. Nature.

[51] Helleday T., Eshtad S., Nik-Zainal S. (2014). Mechanisms underlying mutational signatures in human cancers. Nat. Rev. Genet.

[52] Scully R., Panday A., Elango R., Willis N.A. (2019). DNA double-strand break repair-pathway choice in somatic mammalian cells. Nat. Rev. Mol. Cell Biol..

[53] Chang H.H.Y., Pannunzio N.R., Adachi N., Lieber M.R. (2017). Non-homologous DNA end joining and alternative pathways to double-strand break repair. Nat. Rev. Mol. Cell Biol..

[54] Carloni V., Morganti E., Galli A., Mazzocca A. (2022). The Adaptability of Chromosomal Instability in Cancer Therapy and Resistance. Int. J. Mol. Sci..

[55] Stafylidis C., Vlachopoulou D., Kontandreopoulou C.N., Diamantopoulos P. (2024). Unmet Horizons: Assessing the Challenges in the Treatment of TP53-Mutated Acute Myeloid Leukemia. J. Clin. Med..

[56] Jiang Y., Gao S.J., Soubise B., Douet-Guilbert N., Liu Z.L., Troadec M.B. (2021). TP53 in Myelodysplastic Syndromes. Cancers.

[57] Ebert B.L. (2011). Molecular dissection of the 5q deletion in myelodysplastic syndrome. Semin. Oncol..

[58] Kotsiafti A., Giannakas K., Christoforou P., Liapis K. (2023). Progress toward Better Treatment of Therapy-Related AML. Cancers.

[59] Maciejowski J., de Lange T. (2017). Telomeres in cancer: Tumour suppression and genome instability. Nat. Rev. Mol. Cell Biol..

[60] Davoli T., de Lange T. (2012). Telomere-driven tetraploidization occurs in human cells undergoing crisis and promotes transformation of mouse cells. Cancer Cell.

[61] Maser R.S., DePinho R.A. (2002). Connecting chromosomes, crisis, and cancer. Science.

[62] Negrini S., Gorgoulis V.G., Halazonetis T.D. (2010). Genomic instability—An evolving hallmark of cancer. Nat. Rev. Mol. Cell Biol..

[63] Montoro M.J., Palomo L., Haferlach C., Acha P., Chan O., Navarro V., Kubota Y., Schulz F.I., Meggendorfer M., Briski R. (2024). Influence of *TP53* gene mutations and their allelic status in myelodysplastic syndromes with isolated 5q deletion. Blood.

[64] Yan B., Claxton D., Huang S., Qiu Y. (2020). AML chemoresistance: The role of mutant TP53 subclonal expansion and therapy strategy. Exp. Hematol..

[65] Sallman D.A., Komrokji R., Vaupel C., Cluzeau T., Geyer S.M., McGraw K.L., Al Ali N.H., Lancet J., McGinniss M.J., Nahas S. (2016). Impact of TP53 mutation variant allele frequency on phenotype and outcomes in myelodysplastic syndromes. Leukemia.

[66] Sperling A.S., Guerra V.A., Kennedy J.A., Yan Y., Hsu J.I., Wang F., Nguyen A.T., Miller P.G., McConkey M.E., Quevedo Barrios V.A. (2022). Lenalidomide promotes the development of TP53-mutated therapy-related myeloid neoplasms. Blood.

[67] Papaemmanuil E., Gerstung M., Malcovati L., Tauro S., Gundem G., Van Loo P., Yoon C.J., Ellis P., Wedge D.C., Pellagatti A. (2013). Clinical and biological implications of driver mutations in myelodysplastic syndromes. Blood.

[68] Haferlach T., Nagata Y., Grossmann V., Okuno Y., Bacher U., Nagae G., Schnittger S., Sanada M., Kon A., Alpermann T. (2014). Landscape of genetic lesions in 944 patients with myelodysplastic syndromes. Leukemia.

[69] Malcovati L., Papaemmanuil E., Bowen D.T., Boultwood J., Della Porta M.G., Pascutto C., Travaglino E., Groves M.J., Godfrey A.L., Ambaglio I. (2011). Clinical significance of SF3B1 mutations in myelodysplastic syndromes and myelodysplastic/myeloproliferative neoplasms. Blood.

[70] Walter M.J., Shen D., Ding L., Shao J., Koboldt D.C., Chen K., Larson D.E., McLellan M.D., Dooling D., Abbott R. (2012). Clonal architecture of secondary acute myeloid leukemia. N. Engl. J. Med..

[71] Ding L., Ley T.J., Larson D.E., Miller C.A., Koboldt D.C., Welch J.S., Ritchey J.K., Young M.A., Lamprecht T., McLellan M.D. (2012). Clonal evolution in relapsed acute myeloid leukaemia revealed by whole-genome sequencing. Nature.

[72] Young A.L., Challen G.A., Birmann B.M., Druley T.E. (2016). Clonal haematopoiesis harbouring AML-associated mutations is ubiquitous in healthy adults. Nat. Commun..

[73] da Silva-Coelho P., Kroeze L.I., Yoshida K., Koorenhof-Scheele T.N., Knops R., van de Locht L.T., de Graaf A.O., Massop M., Sandmann S., Dugas M. (2017). Clonal evolution in myelodysplastic syndromes. Nat. Commun..

[74] Abelson S., Collord G., Ng S.W.K., Weissbrod O., Mendelson Cohen N., Niemeyer E., Barda N., Zuzarte P.C., Heisler L., Sundaravadanam Y. (2018). Prediction of acute myeloid leukaemia risk in healthy individuals. Nature.

[75] Abdallah M., Reichard K., Gangat N., Tefferi A. (2024). Treatment-emergent mutations in myelodysplastic syndrome with del(5q)—Lenalidomide related or disease-intrinsic clonal evolution?. Blood Cancer J..

[76] Mack T., Pershad Y., Vlasschaert C., Bejan C.A., Heimlich J.B., Li Y., Mickels N.A., Van Amburg J.C., Ulloa J., Silver A.J. (2025). Germline genetics, disease, and exposure to medication influence longitudinal dynamics of clonal hematopoiesis. Haematologica.

[77] Jadersten M., Karsan A. (2011). Clonal evolution in myelodysplastic syndromes with isolated del(5q): The importance of genetic monitoring. Haematologica.

[78] Cai Z., Kotzin J.J., Ramdas B., Chen S., Nelanuthala S., Palam L.R., Pandey R., Mali R.S., Liu Y., Kelley M.R. (2018). Inhibition of Inflammatory Signaling in Tet2 Mutant Preleukemic Cells Mitigates Stress-Induced Abnormalities and Clonal Hematopoiesis. Cell Stem Cell.

[79] Cong B., Zhang Q., Cao X. (2021). The function and regulation of TET2 in innate immunity and inflammation. Protein Cell.

[80] Sato N., Goyama S., Chang Y.H., Miyawaki M., Fujino T., Koide S., Denda T., Liu X., Ueda K., Yamamoto K. (2024). Clonal hematopoiesis-related mutant ASXL1 promotes atherosclerosis in mice via dysregulated innate immunity. Nat. Cardiovasc. Res..

[81] Min K.D., Polizio A.H., Kour A., Thel M.C., Walsh K. (2022). Experimental ASXL1-Mediated Clonal Hematopoiesis Promotes Inflammation and Accelerates Heart Failure. J. Am. Heart Assoc..

[82] Haase D. (2008). Cytogenetic features in myelodysplastic syndromes. Ann. Hematol..

[83] Zeuthen M., Christoffersen L.S., Hildonen M., Birkedal U., Grubach L., Kjaer L., Skov V., Cowland J.B., Andersen M.K. (2026). Long-read sequencing resolves TP53 allelic status in myeloid neoplasms. Haematologica.

[84] Abel H.J., Mahgoub M., Davarapalli N., Kodgule R., Miller C.A., Fulton R.S., Fronick C., Markovic C., Heath S., Payton J.E. (2025). Evaluation of Long-Read Genome Sequencing for Genomic Profiling of Myeloid Cancers. J. Mol. Diagn..

[85] Haase D., Germing U., Schanz J., Pfeilstocker M., Nosslinger T., Hildebrandt B., Kundgen A., Lubbert M., Kunzmann R., Giagounidis A.A. (2007). New insights into the prognostic impact of the karyotype in MDS and correlation with subtypes: Evidence from a core dataset of 2124 patients. Blood.

[86] Mallo M., Cervera J., Schanz J., Such E., Garcia-Manero G., Luno E., Steidl C., Espinet B., Vallespi T., Germing U. (2011). Impact of adjunct cytogenetic abnormalities for prognostic stratification in patients with myelodysplastic syndrome and deletion 5q. Leukemia.

